# Progress of photoacoustic imaging combined with targeted photoacoustic contrast agents in tumor molecular imaging

**DOI:** 10.3389/fchem.2022.1077937

**Published:** 2022-11-21

**Authors:** Yiwen Zheng, Mengyao Liu, Lixin Jiang

**Affiliations:** Department of Ultrasound, Renji Hospital, School of Medicine, Shanghai Jiaotong University, Shanghai, China

**Keywords:** photoacoustic imaging, targeted contrast agent, molecular imaging, tumor microenvironment, diagnosis

## Abstract

Molecular imaging visualizes, characterizes, and measures biological processes at the molecular and cellular level. In oncology, molecular imaging is an important technology to guide integrated and precise diagnosis and treatment. Photoacoustic imaging is mainly divided into three categories: photoacoustic microscopy, photoacoustic tomography and photoacoustic endoscopy. Different from traditional imaging technology, which uses the physical properties of tissues to detect and identify diseases, photoacoustic imaging uses the photoacoustic effect to obtain the internal information of tissues. During imaging, lasers excite either endogenous or exogenous photoacoustic contrast agents, which then send out ultrasonic waves. Currently, photoacoustic imaging in conjunction with targeted photoacoustic contrast agents is frequently employed in the research of tumor molecular imaging. In this study, we will examine the latest advancements in photoacoustic imaging technology and targeted photoacoustic contrast agents, as well as the developments in tumor molecular imaging research.

## 1 Introduction

With the rapid growth of molecular science and imaging technology in the 1990s, molecular imaging has gradually aroused the interest of biomedical professionals. After the turn of the new century, the National Institutes of Health (NIH) (Zerhouni, 2003) and the National Cancer Institute (Gimi et al., 2005) and other institutions released a series of program announcements and invested significant resources to molecular imaging. The Radiological Society of North America and the Society for Nuclear Medicine and Molecular Imaging defined molecular imaging in 2005 as “molecular imaging techniques that monitor and record the spatial and temporal distribution of molecular or cellular processes for biochemical, biological, diagnostic, and therapeutic applications” (Thakur and Lentle, 2005). Mankoff (2007) further refined the definition of molecular imaging as “ the observation, characterisation, and quantification of biological processes at the molecular and cellular levels of human and other living organisms.” In 2007. In 2008, the World Molecular Imaging Association was founded and the first World Molecular Imaging Congress (WMIC) was held in Nice, France, bringing molecular imaging to the forefront of the minds of researchers.

In oncology, molecular imaging is a crucial tool for guiding integrated and accurate diagnosis and treatment, and it has been utilized in several key areas of cancer diagnosis and treatment, including tumor microenvironment imaging, tumor diagnosis, tumor staging and typing, targeted tumor therapy, and efficacy evaluation. Traditional imaging techniques, such as MRI, CT, ultrasound, etc., rely primarily on the physical properties of tissues (such as density, relaxation, absorption, and scattering) to detect and mark diseases (Weissleder, 1999). However, it is difficult for these techniques to provide information about the molecular composition of tissues, despite the molecular nature of MRI. Few pulse sequences, such as diffusion-weighted imaging (DWI), dynamic contrast-enhanced (DCE), and magnetic resonance spectroscopy, can evaluate particular molecular-level activity without the application of molecular contrast agents (Kircher and Willmann, 2012) (MRS). Positron emission tomography (PET) and single photon emission computed tomography (SPECT) have achieved true molecular imaging through the use of radioactive tracers. However, the application of these two nuclear medicine imaging technologies is still limited by the relatively high cost and the inherent ionizing radiation involved in the imaging process. Photoacoustic imaging is a revolutionary imaging method that combines the excellent resolution of optical imaging with the penetrating depth of acoustic imaging. It is capable of separating the photoacoustic signals of hemoglobin, melanin, fat, water, and other endogenous chromophores based on their distinctive absorption spectra and displaying the spatial distribution of substances on the image *via* post-processing techniques. Non-invasive, real-time response to the metabolic state of a tumor. Photoacoustic imaging method can more accurately represent the distribution of certain molecules when combined with exogenous tailored contrast agents. Photoacoustic imaging combined with targeted photoacoustic contrast agents for tumor molecular imaging will be reviewed in this article.

## 2 Photoacoustic imaging technology

### 2.1 Principle of photoacoustic imaging

The photoacoustic effect was first discovered by Bell (1880) in 1880, and photoacoustic imaging (PAI) is a non-invasive, non-ionizing radiation imaging device developed using this effect. PAI was not brought to the field of imaging biological tissue until the 1970s, when the performance of laser transmitters improved (Kreuzer and Patel, 1971). Since then, advancements have been achieved in instruments, image processing, molecular imaging, and functional imaging. When biological tissues are exposed to a short-pulse laser, chromophores in molecules with light absorption characteristics absorb a certain amount of light energy, causing electrons to jump from a low energy level to an excited state. Since the excited state is not stable, the electrons in the excited state have a tendency to jump back to the ground state, and a portion of the energy released in the transition process is converted into heat energy, The pressure generated by the expansion is transmitted in the form of acoustic wave, which is received by the ultrasonic transducer and then processed to form an image (Ntziachristos and Razansky, 2010).

In terms of excitation wavelength, photoacoustic imaging of biological tissues mostly employs near infrared (NIR) light, which can be further subdivided into near infrared region I (NIR-I, 700–900 nm) and near infrared area II (NIR-II, 1000–1700 nm). NIR-PAI can produce deeper penetration depth, a higher signal-to-noise ratio and more contrast compared to typical visible light biological imaging, which helps to increase spatial resolution (Du et al., 2021). Existing photoacoustic imaging wavelengths are primarily found in the NIR-I window, and longer wavelengths can reduce light scattering, increase image contrast, and reduce the risk of tissue thermal damage. However, due to the lack of imaging contrast agents, imaging research in the NIR-II window is still in its infancy (Wanderi and Cui, 2022).

### 2.2 Main modes of photoacoustic imaging

**FIGURE 1:**
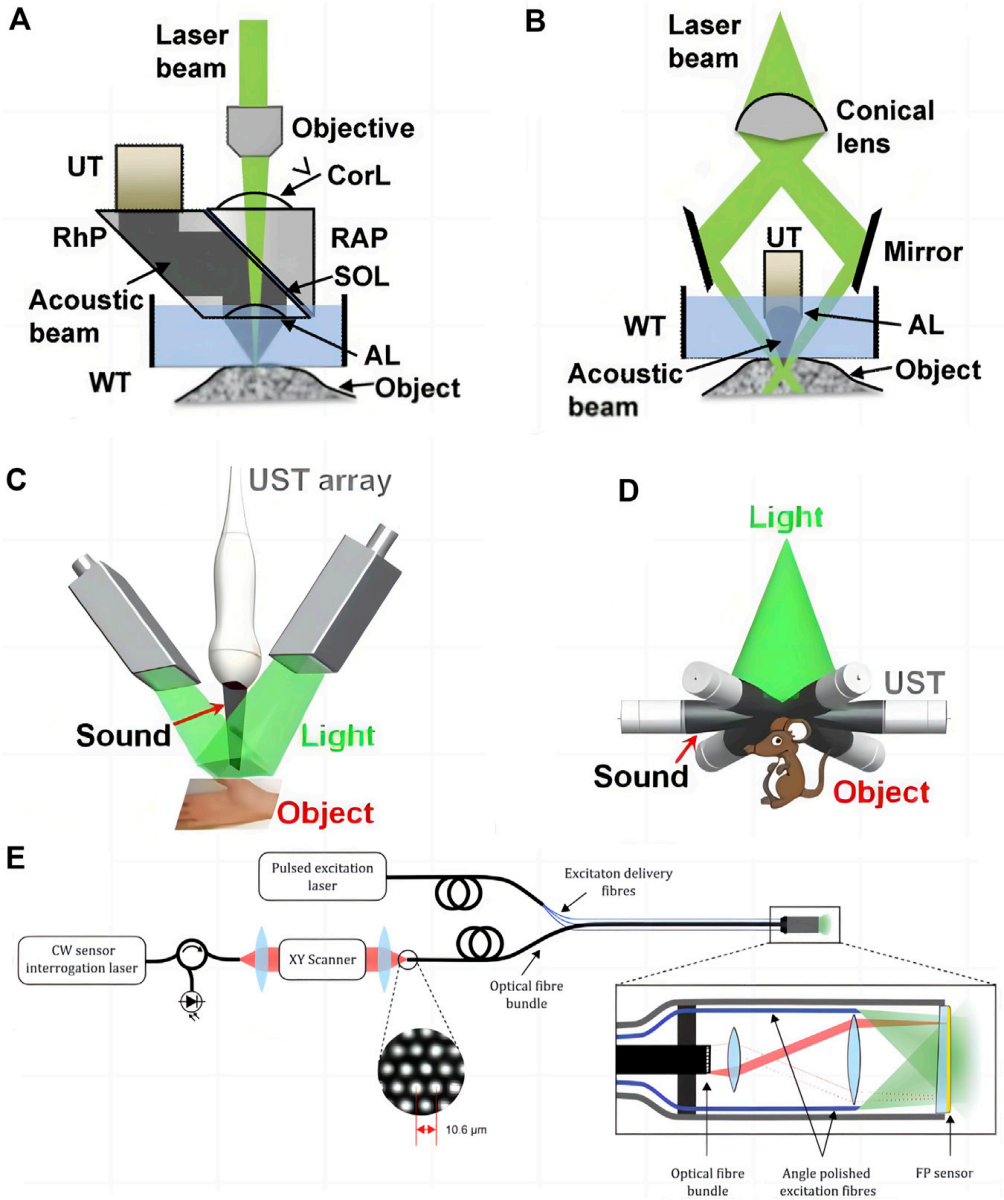
Major embodiments of photoacoustic imaging. **(A)** Second-generation optical-resolution photoacoustic microscopy (G2-OR-PAM). **(B)** Dark-field acoustic-resolution photoacoustic microscopy (AR-PAM). AL, acoustic lens; Corl, correction lens; RAP, right angled prism; RhP, rhomboid prism; SOL, silicone oil layer; UT, ultrasonic transducer; WT, water tank [reproduced from (Yao and Wang., 2013) with permission from John Wiley and Sons]. **(C)** Linear-array PACT. **(D)** Circular-array PACT [reproduced from (Wang and Hu., 2012) with permission from The American Association for the Advancement of Science]. **(E)** Schematic of the flexible all-optical, forward-viewing PA endoscopy probe [reproduced from (Ansari et al., 2020) with permission from The Optical Society].

Researchers have created a range of setups for micro-, meso-, and macro-scale imaging, including organelles, cells, tissues, and organs, based on varied combinations of optical illumination and sound detection techniques (Attia et al., 2019). The main imaging modalities of PAI include photoacoustic microscopy (photo acoustic microscopy, PAM) and photoacoustic tomography (photo acoustic tomography, PAT). N order to achieve intracavity imaging, researchers further developed photoacoustic endoscope (photo acoustic endoscopy, PAE) based on PAM and PAT (Figure 1). Different photoacoustic imaging configurations are essentially a trade-off between resolution and detection depth, and their application scenarios are related to the observed object.

### 2.3 Photoacoustic microscope

During PAM imaging, the focused laser beam is used to excite the target region, and the resultant sound wave is subsequently focused by the acoustic lens and tansferred to the high-frequency ultrasonic transducer. When collecting the information of a large area, because the tissue surface is typically not smooth, large-area data collection requires mechanical conversion, i.e., adjusting the system’s focus according to different areas and obtaining several images that are subsequently combined. Du and Sun (2021) According to the degree of focusing of optical focus and acoustic focus, PAM can be subdivided into optical resolution photoacoustic microscopy (OR-PAM) and acoustic resolution photoacoustic microscopy (AR-PAM). OR-PAM can provide lateral resolution of micron or even submicron scale, which is comparable to optical imaging, however the detection depth is limited to 1–2 mm. AR-PAM employs acoustic focusing and provides a lateral resolution of tens of micrometers, however the attenuation of high-frequency ultrasonic waves still restricts imaging depth to 1–10 mm. Raster scan photoacoustic mesoscopic imaging (RSOM) is an acoustic-resolution photoacoustic microscopy imaging method.

### 2.4 Photoacoustic tomography

During PAT imaging, the unfocused beam is utilized, and the photoacoustic signal is detected by an array of ultrasonic transducers with a scanning function multiple-angle placement. The photoacoustic signals fed back by tissues at different depths come successively at the transducer, and the photoacoustic signals of different tomographic planes are collected by time resolution technology and afterwards reconstructed (Yao and Wang, 2013). PAT further reduces the lateral resolution to several hundred microns since it detects lower frequency ultrasound waves, which are substantially less influenced by acoustic attenuation, and the detection depth can reach 6 cm (Lin and Wang, 2022). It possesses the high resolution of pure optical imaging and high penetration depth of pure acoustic imaging, and has more clinical application potential. Despite the excellent properties of PAI, photoacoustic imaging with a single excitation wavelength is incapable of distinguishing distinct light absorbing components in tissues based on background absorption (Wu et al., 2021). Multiple wavelengths of light are used to highlight the tissue in multispectral photoacoustic tomography (MSOT), which combines multispectral imaging and photoacoustic tomography. In addition, the aliased signals are separated using the characteristic absorption spectra of various compounds. When a three-dimensional volume image is acquired, the acquired information can be used to reconstruct the image through a back projection algorithm, splitting the overlapped spectral data in each voxel, realizing the positioning and quantification of tissue information in a three-dimensional space, and further assigning different chromophores with false colors. Maximal intensity projection (MIP) displays a three-dimensional image of the tissue in real time on three orthogonal x-y, x-z, and y-z planes.

### 2.5 Photoacoustic endoscope

PAE is an endoscopic technique based on PAM and PAT, which is mostly utilized for vascular, digestive and genitourinary examinations. Photoacoustic imaging system incorporates optical fiber, reflector, microlens and ultrasonic transducer into the probe of the endoscope and achieces circular or spiral scanning and signal capture by rotating probe (Jin et al., 2018). Its development and implementation are also contingent upon further technological advances in acoustic coupling and probe downsizing (Guo et al., 2020).

## 3 Photoacoustic imaging contrast agent

### 3.1 Endogenous contrast agent

**FIGURE 2:**
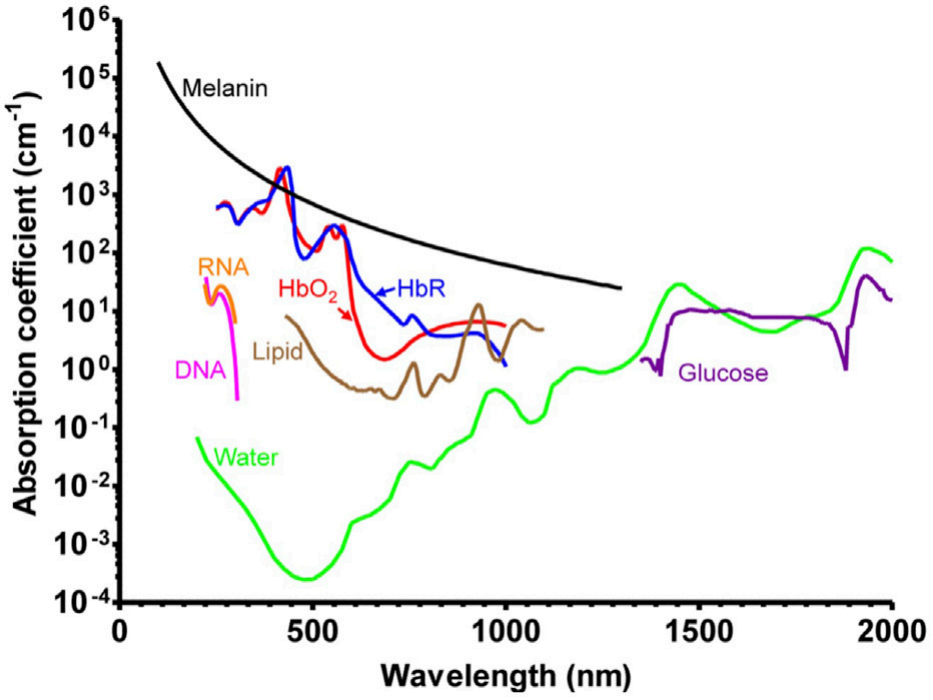
Absorption spectra of major endogenous contrast agents in biological tissue [reproduced from (Wang and Hu., 2012) with permission from The American Association for the Advancement of Science].

Endogenous contrast agents are intrinsic chromophores in biological tissues, primarily hemoglobin (Karlas et al., 2021), melanin (Fan et al., 2014), lipids (Ntziachristos and Razansky, 2010), collagen (Wu et al., 2021), bilirubin (Lee et al., 2017), water (Yan et al., 2021), and DNA and RNA (Yao et al., 2010) (Figure 2). Tumor metabolism and behavior are regulated by intracellular factors and metabolites in the tumor microenvironment (Elia and Haigis, 2021). Changes occur at the molecular level throughout the development of tumors, and the differences in photoacoustic signals created by certain molecules are the basis for the use of endogenous contrast agents in tumor photoacoustic molecular imaging.

The use of endogenous contrast agent imaging offers greater biological safety, can minimize the risk of introducing medcines, and can to some extent reflect the structure and function of tissue. However, the distribution of endogenous contrast agents is not particularly specific. In the NIR-II window, the extinction coefficient of the majority of endogenous contrast agents, with the exception of water, is drastically reduced, and the inherent contrast is sometimes insufficient for imaging (Upputuri and Pramanik, 2020), thereby limiting the detection range of photoacoustic imaging technology. To improve contrast and targeting, exogenous contrast agents are required.

### 3.2 Exogenous contrast agent

Although the endogenous chromophore itself can be eemployed for imaging, the existence of endogenous chromophores can cause interference with the background signal when external contrast agents are applied (MacCuaig et al., 2020). As a contast agent, a material with a high molar extinction coefficient and peak absorption in the near-infrared window is typically required to distinguish the introduced signal from the photoacoustic signal of the tissue. In addition, an ideal exogenous contrast agent must possess the following properties: good photostability, low quantum yield, low toxicity and immunogenicity, high biocompatibility, and high target affinity and specificity (Fu et al., 2019). When creating exogenous contrast agents, there are three compositional components to consider: the substance that may produce photoacoustic signals, the targeting part that can recognize certain markers or biological processes, and the assembly manner of the two (Weber et al., 2016).

### 3.3 Photoacoustic signal

**FIGURE 3:**
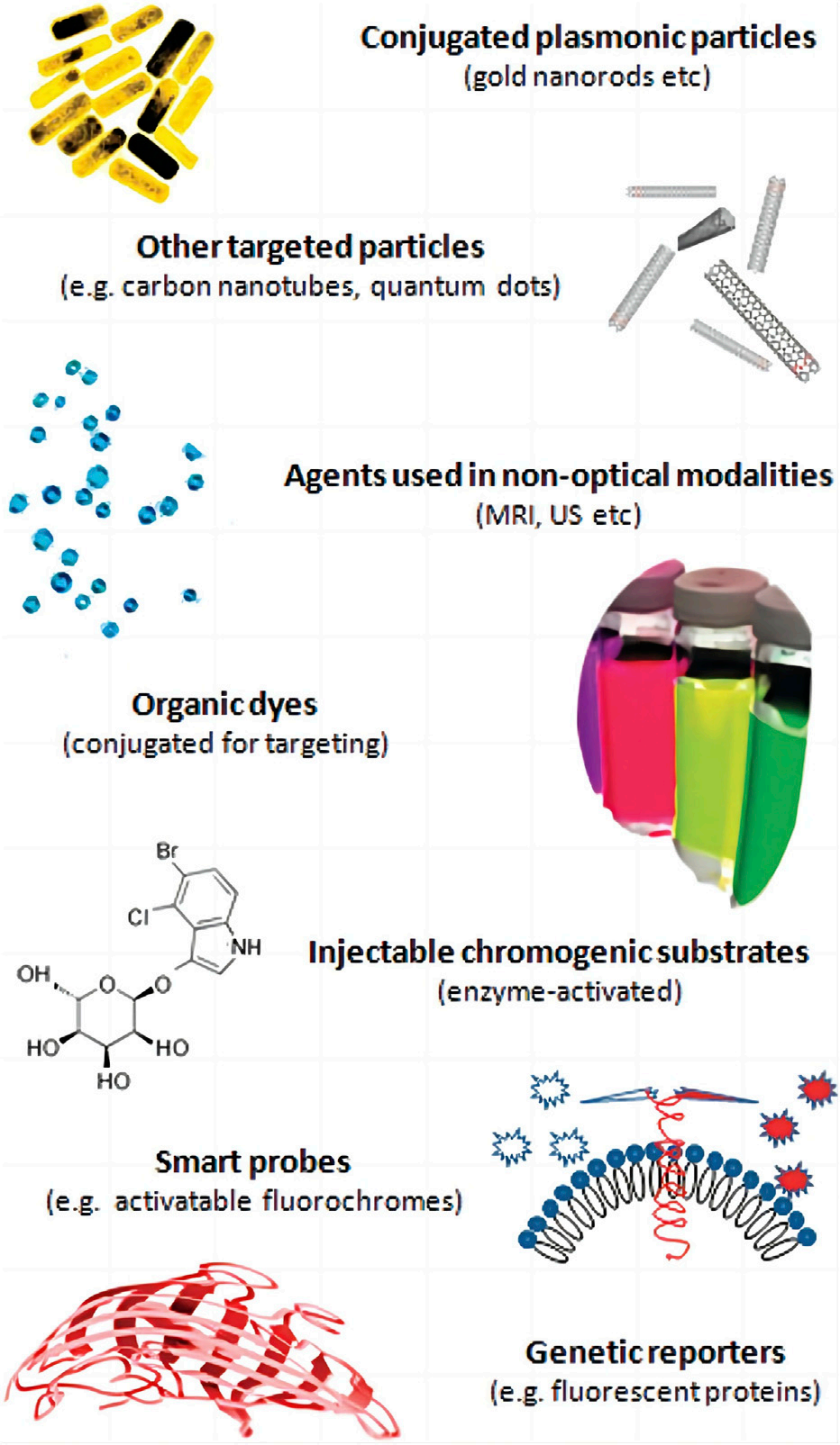
Molecular agents for photoacoustic imaging implementations [reproduced from (Ntziachristos and Razansky., 2010) with permission from American Chemical Society].

Traditionally, photoacoustic signalers have traditionally been split into two categories: organic dyes and nanoparticles (Figure 3).

Small molecule organic dyes were originally used for fluorescence imaging because of their fluorescence properties, and some organic dyes with long conjugated double bonds or ring structures were introduced into the field of photoacoustic imaging because they can balance fluorescence and non-radiative relaxation and achieve a lower quantum yield (Huang and Pu, 2020). In photoacoustic imaging, heptamethine cyanine dyes are a class of small molecule organic dyes exemplified by indocyanine green (ICG). ICG is the first small molecule organic dye approved for clinical use by the US Food and Drug Administration (FDA). It is commonly used in blood flow imaging (Slakter et al., 1995) and has an absorption peak of 780 nm. Using ICG in photoacoustic imaging, (Wang et al., 2004) first reconstructed the distribution of blood vessels in the mouse brain in 2004. Kim et al. (2010) evaluated the photoacoustic signal changes in lymphatic vessels and sentinel lymph nodes before and after ICG injection shortly after ICG its use in cancer research,. In addition, a large number of ICG derivatives have been reported, such as IR825, IR775 and IR780 (Leitão et al., 2020). The Azo dye methylene blue (MB), which has an absorption peak at 664 nm, is another widely used organic dye in clinical settings. Song et al. (2008)) demonstrated the potential of MB for future clinical photoacoustic imaging by identifying sentinel lymph nodes in mice using MB. Squalane (SQ) is an organic dye that has been extensively studied in recent years (An et al., 2014; Ilina et al., 2020). Group-modified squalane is not only soluble in water, but also exhibits characteristic absorption peaks in the NIR window (Umezawa et al., 2008). Small molecular organic dyes are biocompatible and easily eliminated from the body, but free small molecular organic dyes have obvious drawbacks, such as poor water solubility, poor photostability, poor targeting, uncontrolled aggregation, etc. To improve *in vivo* distribution and surface modification, they typically require attachment of proteins, phospholipids, or nanocarriers (Leitão et al., 2020).

Similar to organic dyes, nanoparticles (NPs) are widely used in optical imaging as contrast enhancers prior to their application in photoacoustic imaging; however, the variety and composition of NPs are more complex than those of organic dyes. The composition, dimensions, and geometrical configurations of NPs vary. According to different optical absorption, NPs can also be classified as dye-impregnated particles and particles based on surface plasmon resonance (SPR). The first method employs dyes to increase optical absorption. By modifying the physical characteristics of the particles, the latter can adjust the peak absorption of the near-infrared window (Yang et al., 2009). NPs used in photoacoustic imaging have primarily consisted of metals, carbon materials and organic polymer nanoparticles. Metal particle research focuses on gold nanoparticles. Presently, scientists have synthesized gold nanoparticles with various geometric shapes, including gold nanorods (AuNRs), gold nanospheres (AuNS), and gold nanocavity (AuNCCs), etc. Some gold nanostructures made of hollow can also be used for drug delivery (Bouché et al., 2020). Metal nanoparticles’s toxicity cannot be ignored. These particles can induce oxidative stress within the body after entering it. When they accumulate in the liver, kidney, lung and other organs, they have the potential to affect the activity of metabolic enzymes (Dubey et al., 2015). Carbon nanotubes (CNTs) and other carbon nanoparticles are less toxic because they do not contain metal materials (Schrand et al., 2007). The semiconducting polymer nanoparticles (SPNs) synthesized from bioinert materials also have good biocompatibility, and their flexible composition and function are conducive to modifiying the optical properties and transporting other functional components (Yin et al., 2018). The surface of NPs are in direct contact with tissues, necessitaing surface treatment to achieve targeting, low toxicity and low immunogenicity while maintaining high signal (Lemaster and Jokerst, 2017).

In recent years, in addition to small molecular organic dyes and nanoparticles, a genetically engineered contrast agent has emerged, which uses gene editing technology to alter protein expression, directly generate photoacoustic signals or catalyze the generation of photoacoustic signal chromophores (Du et al., 2021), and has the advantage of long-term original expression. The pathological process of diseases can be observed for an extended period of time at the cellular or molecular level.

### 3.4 Targeting part

Due to the enhanced permeability and retention effect (EPR) in vascular leakage diseases such as tumors, it was previously believed that particles with a diameter of less than 100 nm would accumulate at the tumor site. It forms a “passive targeting” (Melancon et al., 2011), but scholars have recently questioned whether the role of EPR effect in nanoparticle delivery has been overestimated (Sindhwani et al., 2020), and the accumulation and penetration ability of nanoparticles in tumor sites is highly tissue-specific. Therefore, the ideal contrast agent should have a certain degree of active targeting, which requires the use of some target molecules that are overexpressed in tumor tissue and underexpressed in non-target tissue, as well as the use of specific ligand molecules to bind to the target and complete targeted recognition. Small molecules, proteins, aptamers (Kim et al., 2018), polypeptides (Jung et al., 2019), and affibodies (Friedman and St Ståhl, 2009) are examples of common specific ligand molecules. Additionally, targeted therapeutic agents that target tumor-associated antigens or tumor-specific antigens can also be used for targeted diagnosis (Terwisscha van Scheltinga et al., 2011).

### 3.5 Assembly method

There are three primary ways to link the photoacoustic signal to the specific ligand molecule of the target (Tang et al., 2018): one is to use small molecule compounds to directly link the signal molecule and the targeting ligand. Wilson et al. (2018) discovered, using lysine amino NHS ester labeling in conjunction with anti-B7-H3 monoclonal antibody and ICG, that even breast ductal carcinoma *in situ* lesions smaller than 1 mm had significantly higher B7-H3 expression than normal and hyperplastic breast tissue. The althernative is to utilize nanostructures to load or bind signal molecules and to modify nanostructures’ surfaces with targeting ligands. Signaling molecules can be encapsulated in mesoporous silica nanoparticles (MSN), bound to the surface or pores of MSN by covalent bonds, filled in the pores of MSN by physical absorption, or directly doped in the framework of MSN (Sun et al., 2021). After reaching the target area, the synthetic signal molecule-targeting ligand complex is activated by specific biological stimuli, the photoacoustic signal is significantly decreased or increased, and the signal to background ratio (SBR) changes significantly, thereby enhancing the resolution (Du et al., 2021). Yihui (Li et al., 2019) constructed a glutathione-responsive nanoparticle DHP. Fluorescent cyanine dye DiR was encased in hydrophobic core consisting of a disulfide-bound hydroxyethyl starch paclitaxel conjugate (HEES-SS-PTX). Fluorescence of DiR is extinguished when Aggregation induces Quenching (ACQ). But when DHP enters the cancer cell, glutathione cleaves the disulfide bond of HECS-SS-PTX, releasing therapeutic PTX, while DiR recovers its fluorescence.

## 4 Progress in PAI combined with photoacoustic contrast agent research for tumor molecular imaging

### 4.1 Tumor microenvironment imaging

The tumor microenvironment (TME) is comprised of tumor cells, stromal cells, immune cells and extracellular matrix, and it differs significantly from normal tissues in terms of angiogenesis, aerobic respiration and metabolic status. During the growth of solid tumors, the internal angiogenesis is abnormal and dysfunctional (Hanahan and Weinberg, 2011), and the abnormal vasculature affects the delivery of drugs to the target tumor tissue during subsequent therapy. Understanding the blood flow status of tumors, particularly monitoring oxygenation level and vascular density, is essential to elucidate the pathophysiological characteristics of tumors (Klibanov and Hu, 2019) and to assess the potential effects of tumor treatment, especially anti-angiogenic therapy (Bohndiek et al., 2015). Oxyhemoglobin (HbO₂) and deoxyhemoglobin (HbR) each have a wavelength-dependent absorption spectrum between 650 and 950 nm. Photoacoustic imaging technology can quantify the concentration of HbO₂ and HbR in a single blood vessel, calculate the oxygen saturation of blood in a single blood vessel so (Li et al., 2018), and display the vascular structure (Hu and Wang, 2010), calculate the blood flow velocity (Fang et al., 2007), and visualize the growth of atypical blood vessels within the tumor and blood oxygen saturation.

In addition to using endogenous HbO₂ and HbR to analyze the local oxygen supply of tumors, researchers have developed hypoxia-responsive exogenous photoacoustic contrast agents. (Knox et al. (2017) fabricated a hypoxia-targeted contrast agent, HyP-1, with a nitroxide-based hypoxia-responsive trigger on one side and a methoxy substituent on the other, using the highly absorbent and photostable asymmetric Aza-BODIPY dye as the core structure. In a hypoxic environment, Hyp-1 can bind to cytochrome P450 and directly reduce to an aniline structure, resulting in a concentration-dependent intense photoacoustic signal at 770 nm. In addition, photoacoustic imaging can be used to monitor vascular regression and hypoxia following anti-vascular tumor treatment. In BALB/C mice subcutaneously inoculated with 4T1 cells, June (Yang et al., 2019) administered high-dose bevacizumab, low-dose bevacizumab and saline solution, respectively. After 5 days of continuous observation, the signal intensity of HbT and HbR in the tumor region of the treatment group gradually decreased. Photoacoustic imaging’s potential application in the evaluation of anti-vascular tumor therapy was validated.

**FIGURE 4:**
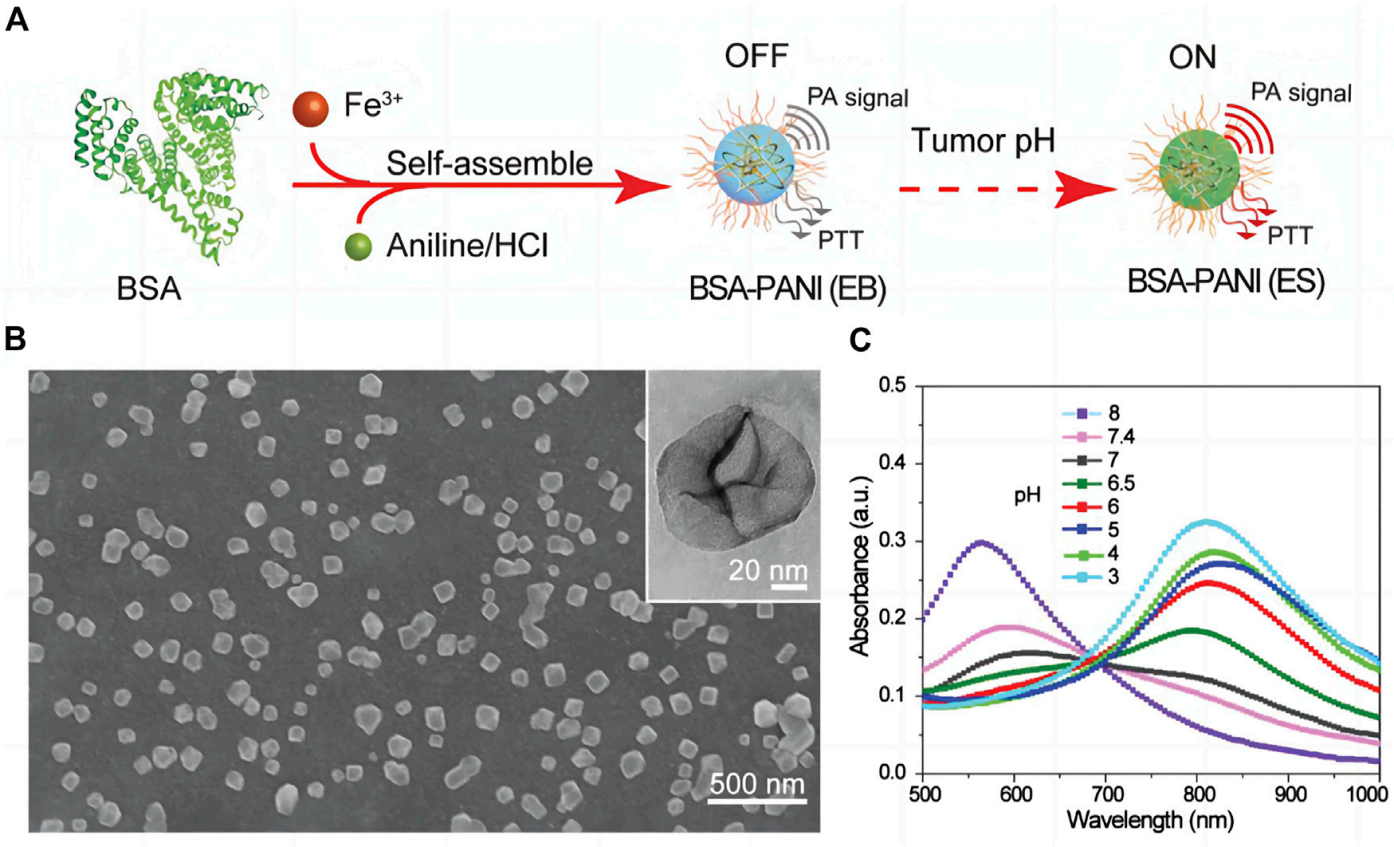
Assembly and characterization of tumor pH-responsive bovine serum albumin (BSA)-polyaniline (PANI) assemblies **(A)** Schematic illustration of the preparation process. **(B)** SEM and TEM (inset) images of the BSA–PANI assembly. **(C)** Absorption spectra of BSA–PANI assemblies dispersed in buffer solutions with different pH values [reproduced from (Tian et al., 2019) with permission from John Wiley and Sons].

The accumulation of lactic acid, hydrogen ions and carbon dioxide in the tumor microenvironment with pH values ranging from 6.5 to 6.8, is a consequence of the metabolism of tumors, which is primarily fueled by glycolysis. Utilizing this property, researchers have designed pH-responsive photoacoustic contrast agents that can image the acidic microenvironment of tumors *in vivo*. Poor stability characterizes photoacoustic contrast agents based on pH-sensitive NIR dyes (Chen et al., 2016). Polyaniline (PANI) is one of the earliest reported organic polymer photothermal agents with good stability and is currently widely used in tumor photoacoustic imaging and photothermal therapy (Li et al., 2015). When pH < 4, PANI can be converted from emerald base (EB) to emerald salt (ES) with high NIR absorbance; however the conversion pH of PANI is lower than that of the tumor microenvironment, necessitating the modification of PANI for tumor photoacoustic imaging. Using the self-doping effect caused by the intermolecular acid-base reaction between the carboxyl group of bovine serum albumin (BSA) and the imine moiety of PANI, (Tian et al., 2019) fabricated a pH-responsive contrast agent based on PANI,. It mediates the transition from EB to ES state at relatively low acidity (pH < 7), thereby amplifying the photoacoustic signal and photothermal effect on tumors (Figure 4). A microenvironment, that is, acidic can also be utilized to deliver nanoparticles. Hua et al. (2021) combined ICG with carboxymethyl chitosan (CS) modified gold nanoparticles to create a multi-level response cluster nanosystem Cs-Au-ICG NPs capable of responding to acidic environment. When the pH value was less than 6.5, the nanoparticles aggregated to form large-size aggregates, and the particle size increased rapidly from 58.8 to 955.1 nm, allowing them to remain in the local tumor. Subsequently, laser irradiation effectively resolved the conflict between the EPR effect and the tumor biological barrier of nanoparticles’ uniform penetration by reducing the aggregates’ size to 5 nm. As a result, the uptake of tumor cells was enhanced, and the conflict between the EPR effect and the tumor biological barrier was effectively resolved.

### 4.2 Diagnosis, staging, and typing of tumors

Molecular imaging technology can directly target tumor cells or specific molecules in cells for identifying imaging, and has distinct advantages for early tumors diagnosis and molecular typing (Lu et al., 2021).

#### 4.2.1 Melanoma

**FIGURE 5:**
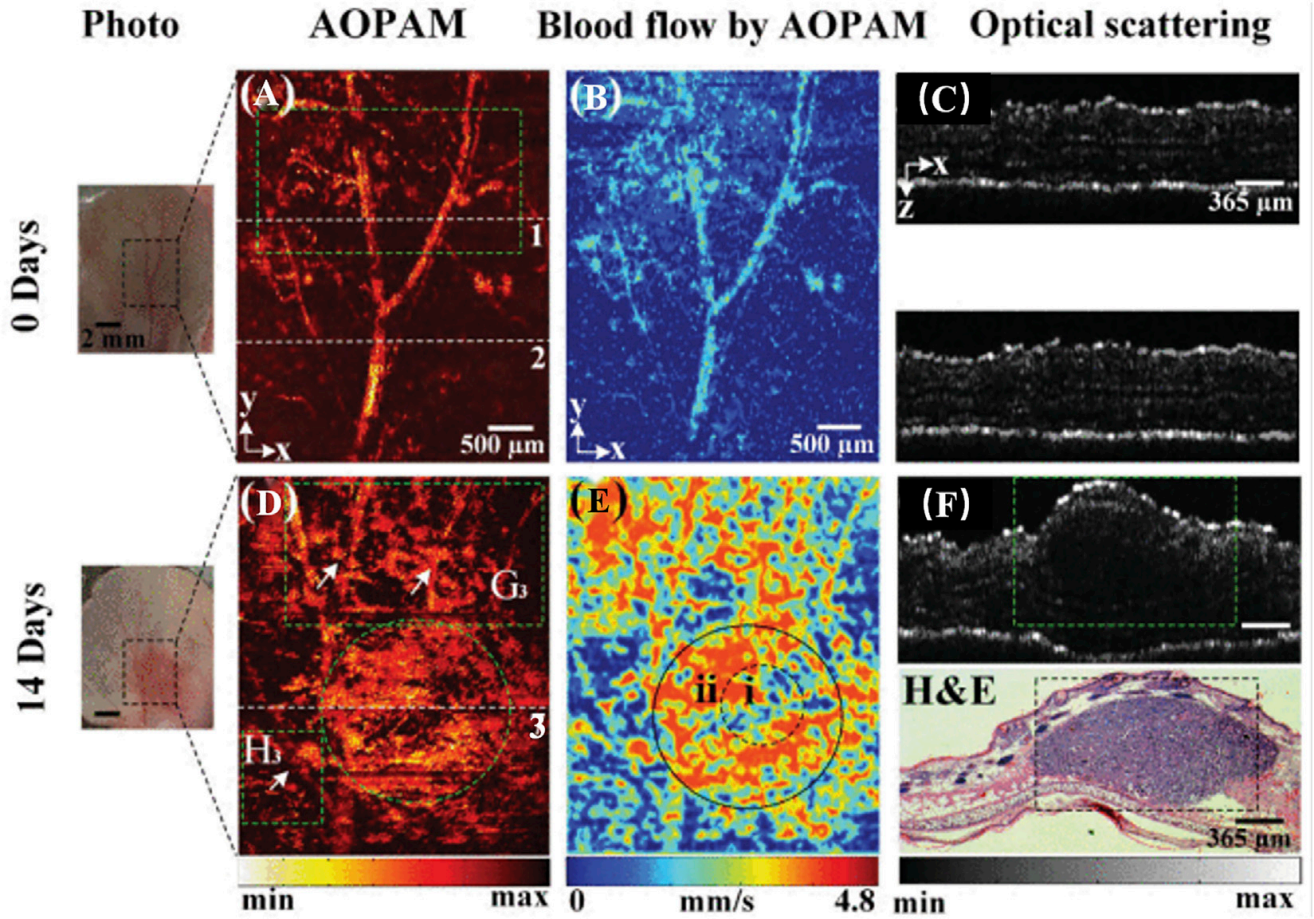
AOPA/OCT images of melanoma progression *in vivo*. **(A,D)** AOPAM optical absorption of intensity images. **(B,E)** Blood flow images by AOPAM. **(C,F)** OCT images corresponding to the white dotted lines 1-3 in **(A,D)**. H&E, hematoxylin and eosin-stained histologic image corresponding to OCT image in **(F)** (reproduced from (Zhou et al., 2020) with permission from IEEE Transactions on Medical Imaging).

In addition to hemoglobin, melanin is a major light absorber in the human body. Melanocytes produce melanin. Malignant melanoma (MM) is one of the most aggressive and deadly skin cancers, and most pathological types of MM continue to produce melanin (Gong et al., 2019). Melanin can detect the depth of invasion of melanoma (Wang et al., 2016) and lymph node metastasis (Grootendorst et al., 2012) to aid in the diagnosis and staging of the disease (Figure 5). Stoffels et al. (2015) used MSOT and organic dye ICG to image lymph nodes of patients with malignant melanoma *in vivo* and *in vitro* to determine whether lymph node metastasis was present. The detection sensitivity was 100% both *in vivo* and *in vitro*, but the detection specificity *in vitro* was only 62.3%. *In vivo* specificity was only 48.6%. Obviously, photoacoustic imaging can help to identify lymph node metastases, but in practice, it is necessary to identify the source of melanin, as melanin is exclusive to malignant melanoma and local melanin photoacoustic signal elevation can also be observed in nevi, melanophils or other pigmented skin lesions. Targeted contrast agents for melanoma markers may aid in reducing the number of false positives. Li et al. (2018) synthesized a dual-mode photoacoustic/ultrasound imaging contrast agent for melanoma-associated antigen (MAGE). In experimental animals, laser irradiation increased the local temperature of the tumor, which promoted the vaporization of perfluorinated hexane encased in the contrast agent and increased the acoustic impedance difference of local tissues for ultrasound detection. It is possible to perform photoacoustic and ultrasound imaging simultaneously. Wu et al. (2020) isolated melanoma cell membrane and wrapped it around hollow copper sulfide nanoparticles loaded with doxorubicin and ICG. These nanoparticles demonstrated effective targeting and ablation of homologous tumor cells in animal studies.

#### 4.2.2 Thyroid cancer

Thyroid nodules are extremely common in clinic, and ultrasound and ultrasound-guided fine needle aspiration biopsy are the most common examination techniques. Using multi-spectral photoacoustic imaging, (Dogra et al., 2014) compared the photoacoustic signal intensity of various endogenous light absorbing groups in benign and malignant nodules as well as thyroid tissue areas. First, it was determined that the signal intensities of HbO₂, HbR, lipid, and water in these three regions varied. In previous research, matrix metalloproteinases (MMPs) have demonstrated some utility in distinguishing benign from malignant thyroid follicular tumors. Using ELISA, Buergy et al. (2009) found that the expression levels of MMP-1 and MMP-9 in thyroid follicular adenocarcinoma tissues were significantly higher than those in adenomas. Cho Mar et al. (2006) reported that MMP-2, MMP-7, and MMP-9 expression was greater in thyroid follicular carcinoma than in thyroid adenoma. Levi et al. (2013) designed the photoacoustic contrast agent Alexa750-CXeeeeXPL GLAGrrrrrXK-BHQ3 to detect MMP in subcutaneously implanted FTC133 thyroid tumors in nude mice. After the contrast agent reached the tumor site and was hydrolyzed by enzymes, it disappeared. Photoacoustic imaging of the tumor was performed using 680 and 750 nm light; the signal acquired at 750 nm was subtracted from the signal recorded at 680 nm; and the photoacoustic signal corresponding to the cleavage of the probe increased significantly.

#### 4.2.3 Ovarian cancer

**FIGURE 6:**
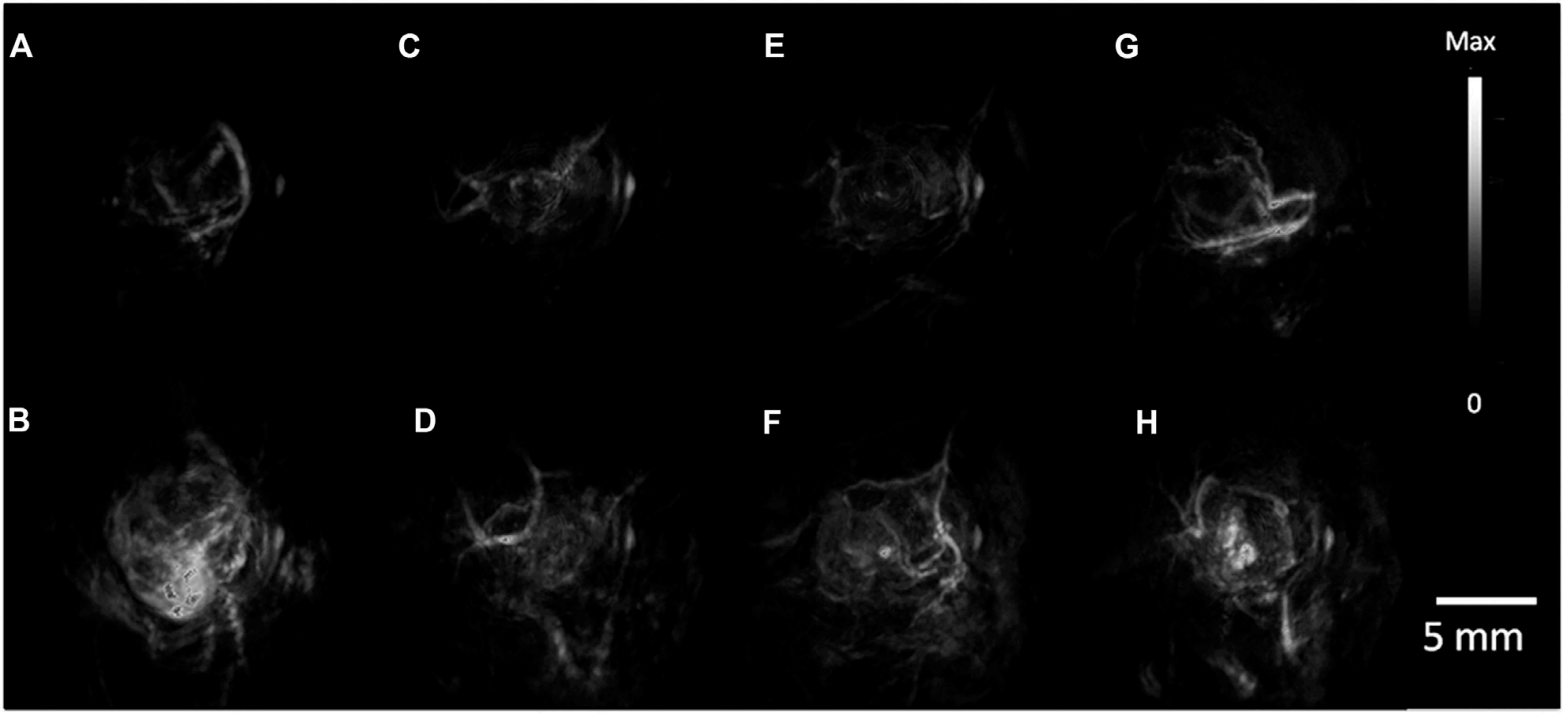
PA Tumor Imaging with passively targeted gold nanorods (GNRs). **(A)** MDA-435S tumors serve as a positive control. Panels **(B–D)**, are 2008, HEY, SKOV3 tumors respectively with global thresholding values before tail-vein injection of 200 ul of 5.4 nM GNR (756 nm resonance) contrast. **(E–H)** are 6 h post injection. PA images were constructed with a volumetric rendering *via* Amide software [reproduced from (Jokerst et al., 2012) with permission from American Chemical Society].

Ovarian cancer has the highest incidence and mortality rate among female reproductive malignancies. The American College of Obstetricians and Gynecologists (ACOG) recommends genetic screening for high-risk women and lists preventive bilateral salpingectomy and oophorectomy as a cancer management strategy (Randall et al., 2017). Although prophylactic resection has been shown to reduce the risk of cancer in women with BRCA 1/2 mutations, it increases the risk of osteoporosis, cardiovascular disease and iatrogenic menopause (Acog Committee Opinion, 2019). Therefore, a more sensitive method for early detection of ovarian cancer is urgently required. In the study of targeted photoacoustic contrast agents for ovarian cancer, the reported contrast agents primarily target folate receptor (FA), epidermal growth factor receptor (HER) (Xi et al., 2014) and tumor microenvironment, and multifunctional contrast agents that can achieve targeted drug delivery, photoacoustic/CT/MR multimodality imaging and synergistic imaging-guided therapy are also research hotspots (Figure 6). Samykutty et al. (2018) encapsulated the organic dye IR780 within a targeted mesoporous silica nanoparticle (V7-RUby) for acidic pH outside ovarian cancer cells. MSOT were used to confirm the targeting of the nanoparticles in mice transplanted with ES-2 and A2780 cells. V7-RUBY containing platinum or paclitaxel resulted in increased tumor cell death in medium with a pH of 6.6, indicating that V7-RUBY may aid in the diagnosis and treatment of ovarian cancer.

#### 4.2.4 Prostate cancer

**FIGURE 7:**
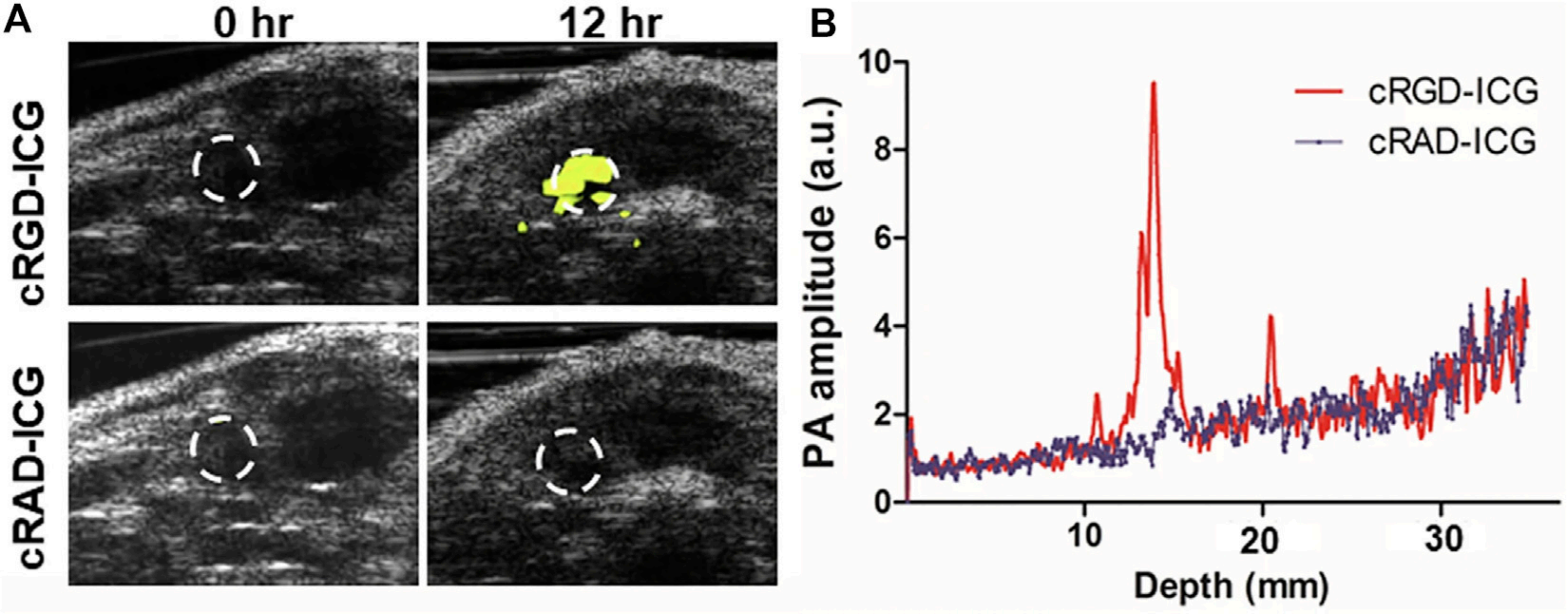
PAI of orthotopic prostate cancer with integrin αvβ3 targeted probes based on ICG (cRGD–ICG). **(A)** *In vivo* integrin αvβ3 PAI images merged with US images. **(B)** PAI signal varied from depths at 12 h [reproduced from (Qiu et al., 2020) with permission from Springer Nature].

Prostate specific antigen (PSA) is a important marker for prostate cancer screening. Patients with abnormally elevated PSA levels typically undergo additional transrectal ultrasound (TRUS) and MRI examinations. If necessary, patients will also undergo a prostate biopsy, which may or may not be successful in removing tumor tissue. Quantified tissue spectral parameters correlated well with prostate Gleason grade in an *in vitro* photoacoustic imaging study of human prostate cancer tissue (Huang et al., 2018). By regulating cell adhesion, proliferation, and migration, α_v_β_3_, a member of the integrin family of cell adhesion molecules, and the arginine-glycine-aspartate sequence (RGD) in the extracellular matrix play a crucial role in the growth, angiogenesis, and metastasis of prostate cancer (Desgrosellier and Cheresh, 2010). Qiu et al. (2020) prepared cRGD-ICG by coupling ICG-NHS with cRGD *via* carboxyl co-reaction, and conducted quantitative analysis in rat orthotopic tumor model (Figure 7). The photoacoustic signal in the tumor region of rats injected with targeted contrast agent was 3.8 times higher than that of rats injected with non-targeted contrast agent (*p* < 0.05). Visualizing and quantifying prostate cancer at the molecular level increases the diagnostic accuracy of tumors.

#### 4.2.5 Breast cancer

In China, breast cancer ranks first in morbidity and fourth in mortality among female malignant tumors. Ultrasound and mammography are the preferred methods of breast examination; breast magnetic resonance imaging can be performed if necessary (He et al., 2021). De Deán-Ben and his colleagues Deán-Ben et al. (2016) were the first to use the MSOT probe for breast imaging using MSOT in the human body. In 2016, the MSOT volume scanning probe was used to scan healthy volunteers with dense glands. The photoacoustic signals of HbR, HbO Hb and melanin were successfully separated, and the volume distribution maps of these endogenous chromophores were reconstructed, demonstrating an excellent capacity to visualize small blood vessels and suggesting that the characteristic absorption peaks of hydroxyapatite in the NIR window may be useful for identifying small calcifications. Diot et al. (2017) subsequently selected 28 excitation wavelengths in the range of 700–970 nm to examine 10 cases of breast cancer, separated the signals of HbR, HbO, lipid, and water, and reconstructed the images. Through endogenous chromophore signaling, anatomical layering is possible. MSOT demonstrated robust angiogenesis around the tumor, a distorted distribution of adjacent fat and water signals, and tumor-feeding arteries. Some lesions exhibited strong HbR signals, indicating that the tumor is hypoxic. This capability to decompose and quantify signals enables MSOT to differentiate benign from malignant breast tumors.

In addition, breast cancer is one of the most in-depth studies of tumor molecular typing. Molecular typing of breast cancer has a variety of methods, the most widely used is the 2013 St. The revised molecular classification of breast cancer as presented at the Gallen International Breast Cancer Conference (Goldhirsch et al., 2011). On the basis of the expression levels of estrogen receptor (ER), progesterone receptor (PR), human epidermal growth factor receptor 2 (HER-2) and proliferation index Ki-67, breast cancer was classified as Luminal A, Luminal B, HER-2 overexpression, triple-negative/basal-like and other subtypes. Beyond the current pathology-based classification, molecular typing can provide clinically relevant information to help understand the biological behavior of breast cancer, guide tumor treatment and determine prognosis (Tsang and Tse, 2020). In a study by Dogan et al. (2019), 2055 breast masses from 1972 women were scanned using a handheld photoacoustic/ultrasound dual-modality imaging probe, and 532 of 653 tumors had immunohistochemical results confirmed by pathology. Among them, 186 were categorized as Luminal a, Luminal B, HER-2 overexpression, triple negative/basal type. Internal and external characteristics of pathologically confirmed cancer were scored by seven readers who were unaware of the pathological results, and the relationship between imaging characteristics and immunohistochemically determined molecular types of tumors was analyzed. Photoacoustic scoring indicators included internal blood vessels and tumor hypoxia, external blood vessels and tumor hypoxia, radial vascular score of marginal zones, and hemoglobin-to-background ratio. Their findings revealed significant differences in photoacoustic signals between Luminal A and B and HER-2 overexpressing and triple-negative/basal-like subtypes, with lower internal scores and higher external scores, indicating that more aggressive subtypes may have more pronounced internal hypoxia, resulting in higher internal characteristic scores (Table 1). Photoacoustic imaging can provide more molecular information than conventional gray-scale ultrasound and aid in molecular typing to a limited extent, but there are few published accouts of it.

**TABLE 1:** Multinomial Logistic Regression Analysis to Show Incremental Benefit of Each Optoacoustic US Feature to Distinguish Luminal Cancers (A and B) from Triple-negative and HER2+ Subtypes in 532 Invasive Cancers (reproduced from (Dogan et al., 2019) with permission from The Radiological Society of North America).

Optoacoustic feature	Evidence of incremental optoacoustic effect (*p*-value) odds ratio*	
Added to all gray-scale US scores^†^		
Internal vessels	0.002^‡^	0.6 (0.5, 0.8)
Internal blush	0.02^‡^	0.7 (0.5, 0.9)
Internal hemoglobin	0.001^‡^	0.6 (0.5, 0.8)
Boundary zone	0.32	0.8 (0.6, 1.1)
Peripheral zone vessels	0.71	0.9 (0.7, 1.2)
Added to sound score only		
Internal vessels	0.002^‡^	0.7 (0.5, 0.8)
Internal blush	0.016^‡^	0.7 (0.5, 0.9)
Internal hemoglobin	0.002^‡^	0.7 (0.5, 0.8)
Boundary zone	0.78	0.9 (0.7, 1.2)
Peripheral zone vessels	0.25	1.1 (0.9, 1.4)

Note—Data in parentheses are 95% confidence intervals. HER2+ = human epidermal growth factor receptor 2/neu positive.

*Odds ratios represent the exponentiated regression classification coefficients.

^†^All five gray-scale US, terms were included in the model: shape, internal texture, sound, boundary zone, and peripheral zone.

^‡^ *p*-Value less than 0.05 is indicative of statistical significance.

## 5 Conclusion and perspective

Molecular imaging is one of the essential precision medicine tools. Photoacoustic imaging as a new imaging technology in recent years, with the improvement of imaging equipment and the optimization of image reconstruction algorithm, the application scenarios of photoacoustic imaging are gradually aligning with clinical practice; when combined with targeted photoacoustic contrast agents, it is useful for non-invasive and dynamic display of tumor-related characteristics. Enhance the capacity to diagnose, treat and assess the disease prognoses. Nevertheless, photoacoustic imaging has some inherent drawbacks. First, there is substantial heterogeneity between different parts of the human body, and additional research is required to clarify how to interpret photoacoustic signals from different parts and depths. Secondly, despite the fact that a large number of photoacoustic contrast agents have emerged this year, the development, preparation and clinical transformation of targeted photoacoustic contrast agents present numerous challenges. In order to achieve the ultimate clinical transformation, it will be necessary for future research to optimize the physical and chemical properties of contrast agents, enhance their biological safety, and enhance their targeting.
