# Corrigendum: Progress of photoacoustic imaging combined with targeted photoacoustic contrast agents in tumor molecular imaging

**DOI:** 10.3389/fchem.2022.1121672

**Published:** 2022-12-21

**Authors:** Yiwen Zheng, Mengyao Liu, Lixin Jiang

**Affiliations:** Department of Ultrasound, Renji Hospital, School of Medicine, Shanghai Jiaotong University, Shanghai, China

**Keywords:** photoacoustic imaging, targeted contrast agent, molecular imaging, tumor microenvironment, diagnosis

In the original article, the **Funding** statement was missing. The correct **Funding** statement is as follows:

“The authors greatly acknowledge the financial support from the National Natural Science Foundation of China (Grant No. 81771850, 82171936).”

The authors apologize for this error and state that this does not change the scientific conclusions of the article in any way. The original article has been updated.

